# Predictors of allergen sensitization in Singapore children from birth to 3 years

**DOI:** 10.1186/s13223-016-0161-x

**Published:** 2016-10-24

**Authors:** Evelyn Xiu Ling Loo, Jordan Zheng Ting Sim, Anne Goh, Oon Hoe Teoh, Yiong Huak Chan, Seang Mei Saw, Kenneth Kwek, Peter D. Gluckman, Keith M. Godfrey, Hugo Van Bever, Yap Seng Chong, Bee Wah Lee, Michael S. Kramer, Lynette Pei-chi Shek

**Affiliations:** 1 Singapore Institute for Clinical Sciences (SICS), Agency for Science, Technology and Research (A*STAR), Singapore, Singapore; 2 National University of Singapore, Singapore, Singapore; 3 Allergy Service, Department of Paediatrics, KK Women’s and Children’s Hospital Singapore, Singapore, Singapore; 4 Respiratory Service Medicine, Department of Paediatrics, KK Women’s and Children’s Hospital, Singapore, Singapore; 5 Biostatistics Unit, Yong Loo Lin School of Medicine, National University Health System, Singapore, Singapore; 6 Saw Swee Hock School of Public Health, National University of Singapore, Singapore, Singapore; 7 Department of Maternal Fetal Medicine, KK Women’s and Children’s Hospital (KKH), Singapore, Singapore; 8 Growth, Development and Metabolism Programme, Singapore Institute for Clinical Sciences (SICS), Agency for Science, Technology and Research (A*STAR), Singapore, Singapore; 9 Liggins Institute, University of Auckland, Auckland, New Zealand; 10 NIHR Southampton Biomedical Research Centre, University of Southampton and University Hospital Southampton NHS Foundation Trust, Southampton, SO16 6YD UK; 11 Medical Research Council Lifecourse Epidemiology Unit, Southampton, SO16 6YD UK; 12 Department of Paediatrics, Yong Loo Lin School of Medicine, National University of Singapore, 1E Kent Ridge Road, Level 12, NUHS Tower Block, Singapore, 119228 Singapore; 13 Khoo Teck Puat-National University Children’s Medical Institute, National University Hospital, National University Health System, Singapore, Singapore; 14 Department of Obstetrics and Gynaecology, Yong Loo Lin School of Medicine, National University of Singapore, Singapore, Singapore; 15 Department of Epidemiology, Biostatistics and Occupational Health, McGill University Faculty of Medicine, Montreal, Canada; 16 Department of Pediatrics, McGill University Faculty of Medicine, Montreal, Canada

**Keywords:** Predictors, Allergen sensitization, Eczema, Skin barrier, Wheeze

## Abstract

**Background:**

Immune responses in allergic diseases begin with allergen sensitization, which usually occurs in childhood. Allergen sensitization involves a complex interplay of genetic and environmental factors, and sensitization patterns may change with age.

**Objective:**

To determine the predictors of allergen sensitization in the first 3 years of life in the growing up in Singapore towards healthy outcomes (GUSTO) prospective birth cohort study.

**Methods:**

Interviewers collected information on demographics, family history of allergy, social and lifestyle factors, and the child’s health. We analyzed data from 849 children who completed skin prick testing (SPT) to inhalant allergens (house dust mites: *Dermatophagoides pteronyssinus*, *Dermatophagoides farinae,* and *Blomia tropicalis*) and food allergens (egg, peanut and cow’s milk) to assess risk factors for allergen sensitization at 18 months. To ensure that clinical phenotypes preceded allergen sensitization, we also analyzed data from 649 children who had a negative skin prick test at 18 months and completed skin prick testing at 36 months.

**Results:**

We observed a significant association between eczema reported before 18 months and a positive SPT at 18 months [aOR 4.5 (1.9–10.7)]. Ninety-five (14.6 %) children with negative SPTs at 18 months developed positive tests at 36 months. Onset of eczema before 18 months was associated with an increased risk of new allergen sensitization at 36 months among children non-sensitized at 18 months [aOR 3.4 (1.2–9.3)]. An association was seen between wheeze reported before 18 months and new allergen sensitization at 36 months [aOR 3.2 (1.1–9.1)]. We found no significant association, however, between rhinitis reported before 18 months and new allergen sensitization at 36 months.

**Conclusions:**

Early onset of eczema and wheeze are risk factors for later allergen sensitization, suggesting a possible increased susceptibility to allergen exposure through an impaired skin barrier or defective airway epithelium.

*Trial registration* NCT01174875 Registered 1 July 2010, retrospectively registered

## Background

Immune responses in allergic diseases begin with allergen sensitization. Allergen sensitization involves a complex interplay of genetic and environmental factors, and sensitization patterns have been reported to change with age [1–3]. A cross-sectional study on the sensitization pattern in Singapore was conducted more than 10 years ago in a group of 75 children below the age of 3 years who have symptoms suggestive of atopic disorders [4]. In this group of children, the prevalence of cow’s milk sensitization was the highest at 45.9 %, followed by egg white at 38.7 % and dustmites (31.4 % to *Dermatophagoides pteronyssinus*, and 25.5 % to *Blomia tropicalis*) [4].

Given the changing environment, we evaluated risk factors of allergen sensitization in the first 3 years of life among Singapore children participating in a birth cohort study. To our knowledge, this is the first prospective study in Asia to assess risk factors for allergen sensitization in the first 3 years of life.

In addition, allergic diseases such as eczema, rhinitis and wheeze that occur in early childhood may be without allergen sensitization [5]. However, knowledge about the association between early clinical phenotypes and the development of subsequent allergen sensitization is limited, as most studies have not assessed non-sensitized children, but have instead analyzed the association between early allergen sensitization and the subsequent development of allergic diseases [6, 7]. The aim of this study is to study the associations between the clinical phenotypes and the development of allergen sensitization in the first 3 years of life. We hypothesized that clinical phenotypes may precede and predict the development of allergen sensitization.

## Methods

### Questionnaires

The methodology of the GUSTO growth cohort study has been previously described [8, 9]. Briefly, we recruited healthy pregnant mothers who agreed to the follow-up of their offspring. Interviewers gathered information on demographics, family history of allergy, social and lifestyle factors and the child’s health. Definitions were standardized in questionnaires administered at 3 weeks and 3, 6, 9, 12, 15, 18, 24 and 36 months and based on the modified International Study of Asthma and Allergies in Children (ISAAC) questionnaire to ensure consistency during interviews and home visits. Presence of eczema was based on the answer to the written question: “Has your child ever been diagnosed with eczema?” “Wheezing” was based on a positive answer to the written question “Has your child ever wheezed?”, while “rhinitis” was based on a positive response to the question “Has your child ever had sneezing, running nose, blocked or congested nose, snoring or noisy breathing during sleep or when awake that has lasted for 2 or more weeks duration?” Study team members telephoned the mothers who reported rhinitis to collect information on the number of episodes of rhinitis and the duration of rhinitis. A case prior to 18 months required a single episode that lasted for at least 4 weeks or two or more episodes each lasting at least 2 weeks. New cases of rhinitis after 18 months were defined by one or more episodes lasting at least 2 weeks.

Classification of breastfeeding has been previously described [10]. High breastfeeding was defined by exclusive or predominant breastfeeding for at least 4 months, with subsequent partial breastfeeding to at least 6 months, while low breastfeeding was defined as exclusive formula feeding or weaning before 3 months. Intermediate breastfeeding was defined as breastfeeding to at least 3 months but without meeting the criteria for high breastfeeding.

### Skin prick testing

Skin prick testing (SPT) to inhalant allergens (house dust mites *D. pteronyssinus*, *Dermatophagoides farinae,* and *B. tropicalis*) and food allergens (egg, peanut and cow’s milk) was carried out at both 18–36 months. All of the allergens for skin prick testing were obtained from Greer Laboratories (Lenoir, NC, USA), except for *B. tropicalis*, which was obtained from our laboratory. *B. tropicalis* extract was prepared as previously described [11]. Briefly, liquid nitrogen frozen cultured mites were ground in a mortar. Protein extraction was performed using phosphate-buffered saline(PBS) containing 1 mM phenylmethylsulphonyl fluoride (Sigma) and 1 mM EDTA at 4 °C overnight. The extract was filtered through 0.22 mm membrane and quantified by Pierce™ BCA Protein Assay (ThermoFisher), 200 μg/ml of crude mite extract in 50 % glycerol buffer was prepared.

All tests were interpreted as positive if the wheal was at least 3 mm, and a child was considered as SPT-positive if any one or more of the individual tests was positive with a positive reaction to histamine (positive control) and a negative reaction to saline (negative control).

### Study populations

We carried out separate analyses in two study populations. The first comprised all children who had completed skin prick testing at 18 months and was used to assess predictors of allergen sensitization at 18 months. The second (a subpopulation of the first) comprised children with negative skin prick tests for all allergens at 18 months and was used to analyze clinical phenotypes that affected the subsequent development of new allergen sensitization at 36 months.

### Statistical analysis

Statistical analysis was carried out using SPSS version 20.0 (IBM SPSS Statistics, Armonk, NY). The differences in demographic characteristics between the study populations were tested by Chi-square tests. The *p* value represents an overall comparison among the 3 groups, in terms of the demographic variable. A p value <0.05 indicates that there is a difference among the 3 groups for the demographic variable. Associations between antecedent predictors and subsequent allergen sensitization were estimated using multivariable logistic regression adjusting for sex, ethnicity, maternal education, family history of allergy, mode of delivery (vaginal vs cesarean), breastfeeding, antibiotic consumption in the first 6 months of life, dog ownership, living near an expressway, and exposure to tobacco smoke in the home at 12 months. These confounders were selected by a review of the literature [12–14].

## Results

Of the 1247 subjects recruited into GUSTO, 849 completed skin prick testing at 18 months and comprised the first study population. Of the 849 subjects, 477 (56.2 %) were Chinese, 440 (51.8 %) were male and 505 (59.5 %) were born to mothers with at least 12 years of education. No significant differences in these characteristics were observed between subjects who completed the skin prick testing and those who did not (data not shown). A total of 649 children had a negative SPT at 18 months and completed a second SPT at 36 months. Of these, 367 (56.5 %) were Chinese, 335 (51.6 %) were male and 380 (58.6 %) were born to mothers with at least 12 years of education.

Table 1 compares the characteristics of children who remained unsensitized at both 18–36 months, those sensitized at 18 months and those with new sensitization at 36 months.

**Table 1:** Comparison of study groups

Demographics	Never sensitized at both M18 and M36	Sensitized at M18	New sensitization at M36 among children non-sensitized at M18	p value
	n (%)	n (%)	n (%)	
Sex				
Female	270 (48.7)	48 (41.7)	43 (45.7)	0.4
Male	284 (51.3)	67 (58.3)	51 (54.3)	
Ethnicity				
Chinese	305 (55.1)	66 (57.4)	62 (65.3)	0.049
Malay	147 (26.5)	38 (33.0)	19 (20.0)	
Indian	102 (18.4)	11 (9.6)	14 (14.7)	
No family history of allergy	184 (51.1)	35 (43.8)	24 (40.7)	0.2
Family history of allergy	176 (48.9)	45 (56.2)	35 (59.3)	
Vaginal delivery	385 (69.5)	77 (67.0)	74 (79.6)	0.1
Non-vaginal delivery	169 (30.5)	38 (33.0)	19 (20.4)	
Low breastfeeding	242 (45.2)	37 (33.9)	26 (29.2)	<0.01
Intermediate breastfeeding	227 (42.4)	55 (50.5)	55 (61.8)	
High breastfeeding	66 (12.3)	17 (15.6)	8 (9.0)	
No antibiotic consumption in the first 6 months	437 (84.2)	98 (89.1)	76 (88.4)	0.3
Antibiotic consumption in the first 6 months	82 (15.8)	12 (10.9)	10 (11.6)	
No dog ownership at 12 months	281 (92.7)	55 (94.8)	57 (98.3)	0.3
Dog ownership at 12 months	22 (7.3)	3 (5.2)	1 (1.7)	
Not staying near an expressway at 12 months	252 (83.4)	43 (74.1)	43 (74.1)	0.1
Staying near an expressway at 12 months	50 (16.6)	15 (25.9)	15 (25.9)	
No exposure to smoke at 12 months	196 (64.3)	43 (72.9)	42 (72.4)	0.3
Exposure to smoke at 12 months	109 (35.7)	16 (27.1)	16 (27.6)	
Maternal education levels ≤12 years	232 (42.2)	43 (37.7)	31 (33.3)	0.2
Maternal education levels >12 years	318 (57.8)	71 (62.3)	62 (66.7)	

### Prevalence of allergen sensitization at 18–36 months

The positive SPT rate increased from 115 (13.5 %) at 18 months to 200 (23.4 %) at 36 months. Similarly, the positive SPT rate to dust mites increased from 95 (11.2 %) at 18 months to 194 (22.6 %) at 36 months. In contrast, positive SPT to one or more food allergens decreased from 38 (4.5 %) at 18 months to 18 (2.1 %) at 36 months.

At 18 months, 95 subjects (11.2 %) showed a positive SPT to one or more house dust mite: 71 (8.4 %) to *D. pteronyssinus*, 57 (6.7 %) to *D. farinae* and 8 (0.9 %) to *B. tropicalis*; 38 (4.5 %) subjects had a positive SPT to food allergens: 6 (0.7 %) to cow’s milk, 11 (1.3 %) to peanut and 30 (3.5 %) to egg. Out of these 38 subjects, 11 had reported clinical food allergies: 1 to cow’s milk, 1 to egg and peanut, 8 to egg and 1 to peanut. These subjects with clinical food allergies also have supportive SPT data.

Ninety-six (14.7 %) children with negative SPTs at 18 months developed positive tests at 36 months: 92 subjects (14.2 %) to one or more dust mites, 76 (11.8 %) to *D. pteronyssinus*, 58 (8.9 %) to *D. farinae* and 9 (1.4 %) to *B. tropicalis*. New food allergen sensitization was observed to peanut in 3 (0.5 %) and to egg in 1 (0.2 %). However these subjects with new food sensitizations did not report clinical symptoms of food allergies.

### Predictors of allergen sensitization at 18 months

We found a significant association between eczema reported before 18 months and a positive SPT at 18 months [aOR 4.5 (1.9–10.7), Table 2] after adjusting for sex, ethnicity, maternal education, family history of allergy, mode of delivery, breastfeeding, antibiotic consumption in the first 6 months of life, owning a dog, living near an expressway and exposure to tobacco smoke at 12 months of life. There was also a significant association between rhinitis reported before 18 months and a positive SPT [aOR 2.8 (1.1–7.3), Table 2] after adjusting for confounders. No significant associations were observed between wheeze reported before 18 months and allergen sensitization at 18 months. Further analysis showed that eczema reported before 18 months was significantly associated with both inhalant allergen sensitization [aOR 3.7 (1.5–9.4)] and food allergen sensitization [aOR 7.6 (1.9–30.7)] (data not shown). No significant associations were observed between wheeze or rhinitis reported before 18 months and inhalant or food allergen sensitization at 18 months.

**Table 2:** Findings in multivariable analyses of associations between reported early allergic conditions and positive SPT at 18 months among GUSTO children

Risk factor	N (%)	Adjusted^a^ OR (95 % CI)
Rhinitis reported before 18 months of life	24 (25.5)	*2.8 (1.1–7.3)*
Wheeze reported before 18 months of life	29 (29.0)	1.9 (0.8–4.5)
Eczema reported before 18 months of life	40 (41.7)	*4.5 (1.9–10.7)*

Adjusted OR in italics have a p value less than 0.05

^a^Adjusted for sex, ethnicity, maternal education levels, family history of allergy, vaginal delivery, breastfeeding, antibiotic consumption in the first 6 months of life, dog ownership, staying near an expressway, exposure to smoke at 12 months

### Predictors of new cases of allergen sensitization at 36 months

Eczema reported before 18 months [aOR 3.4 (1.2–9.3)] was associated with an increased risk of new allergen sensitization at 36 months among children with negative SPTs at 18 months (Table 3). An association was also observed between wheeze reported before 18 months and new allergen sensitization at 36 months [aOR 3.2 (1.1–9.1)]. Although we found no significant association between rhinitis reported before 18 months and new allergen sensitization at 36 months, rhinitis first reported after 18 months increased the risk of development of allergen sensitization at 36 months [aOR 3.0 (1.05–8.3), Table 3]. For subjects with onset of rhinitis in the first 18 months, 25.7 % had positive SPT to house dust mites at 36 months. For subjects with onset of rhinitis after 18 months, 31.5 % had a positive SPT to house dust mites at 36 months. No significant associations were observed between onset of wheeze or eczema first reported after 18 months and allergen sensitization at 36 months. Separate analysis of inhalant allergen and food allergen sensitization showed a significant association between wheeze reported before 18 months and new inhalant sensitization at 36 months [aOR 3.2 (1.1–9.1)] and a borderline association between eczema reported before 18 months and new inhalant sensitization at 36 months [aOR 2.9 (1.0–8.3), p = 0.052] (data not shown). Rhinitis first reported after 18 months also increased the risk of developing new inhalant sensitization at 36 months [aOR 2.9 (1.05–8.3)] while no significant associations were seen between onset of wheeze and eczema first reported after 18 months and new inhalant allergen sensitization at 36 months. The number of subjects with new food allergen sensitization at 36 months was too small to analyze.

**Table 3:** Findings in multivariable analyses of associations between reported allergic conditions and positive SPT at 36 months among GUSTO children with negative SPT at 18 months

Risk factor	N (%)	Adjusted^a^ OR (95 % CI)
Rhinitis reported in the first 18 months	8 (11.3)	1.3 (0.3–5.5)
*Wheeze reported in the first 18* *months*	29 (36.7)	*3.2 (1.1–9.1)*
*Eczema reported in the first 18* *months*	17 (21.0)	*3.4 (1.2–9.3)*
*Rhinitis first reported after the first 18* *months*	23 (25.8)	*3.0 (1.05–8.3)*
Wheeze first reported after the first 18 months	10 (11.0)	1.0 (0.2–3.9)
Eczema first reported after the first18 months	4 (4.8)	0.5 (0.1–4.3)

Adjusted OR in italics have a p value less than 0.05

^a^Adjusted for sex, ethnicity, maternal education levels, family history of allergy, vaginal delivery, breastfeeding, antibiotic consumption in the first 6 months of life, dog ownership, staying near an expressway, exposure to smoke at 12 months

## Discussion

Similar to the changes we observed in sensitization, other cohort studies have also noted a marked increase over the first 6 years of life in sensitization to inhalant allergens: from 1.5 to 26 % in Germany [15] and 4–21 % in Korea [16]. The Korean study also reported a decrease in food allergen sensitization from 10 to 2 % [16]. These consistent and opposite changes in sensitization patterns with age for inhalant and food allergens suggest that they may be independent of genetic and environmental factors.

Our observation that eczema was a predictor for allergen sensitization at 18 months suggests that allergen sensitization may occur through an impaired skin barrier. Stronger support for this suggestion is the fact that eczema preceded later allergen sensitization among children not sensitized at 18 months. This combination of findings leads us to speculate that sensitization to allergens may have occurred through an impaired (eczematous) skin barrier in early life [17]. Of note, however, eczema with onset after 18 months was not associated with new allergen sensitization at 36 months.

Although eczema being a risk factor for allergen sensitization has been reported, however knowledge on this in Asian countries is limited where genetic constitution differs from the European countries, hence we performed this analysis in an Asian prospective birth cohort study. In agreement with our observations, the Melbourne Atopy Cohort Study, which recruited 552 at-risk children, also showed that children with eczema in the first 6 months of life had elevated risk of developing new allergen sensitization at 1–2 years and proposed that sensitization may have occurred through a dysfunctional skin barrier [17]. Further evidence of the contribution of early-onset eczema to later allergen sensitization was provided by the Childhood Asthma Prevention Study (CAPS) in Australia, which followed more than 500 high-risk children from birth to 5 years. That study found that eczema was an independent predictor of allergen sensitization by the age of 5 years among children who were not sensitized at 18 months [18]. Another study from Sweden reported that 80 % of children with eczema developed later sensitization to inhalant allergens [19]. Besides this, in another study of 373 high risk infants, persistent early onset eczema increased the risk of both aeroallergen and food allergen sensitization at 7 years [20]. Another German cohort study (n = 1314) also found that atopic dermatitis in first 3 months of life was a risk factor for aeroallergen sensitisation at 5 years [21].

Early eczema may also increase the risk of later food allergies. A study of 3-month-old infants from England and Wales reported that early onset of eczema was associated with subsequent sensitization to foods [22]. Helen et al. [23] found that exposure to environmental peanut antigen in dust was a risk factor for later positive peanut SPT; the odds increased in children with eczema and increased even further in children with severe eczema.

Other studies have reported similar results with other allergic outcomes. A study from Sweden that followed 3124 children to 5 years reported that onset of eczema in the first year of life was associated with increased risk of developing asthma and rhinitis, while later onset of eczema was not [24]. De Benedetto [25] reviewed data suggesting that several epidermal alarmins may lead to a Th2 immune response when the skin barrier is compromised. Moreover, transient defects in the skin barrier may provide signals that lead to movement of Langerhans dendritic cells through tight junctions and engulfment of antigens present on the skin surface. Animal work also suggests that allergens such as house dust mites and food allergens can activate antigen-presenting cells in the epidermis, leading to allergen sensitization and allergic diseases [26]. Besides this, mutations in the filaggrin gene and filaggrin deficiency are known to increase the risk of development of eczema [27, 28] as filaggrin, an epidermal protein plays an essential role in maintaining the integrity of the epidermis by forming the corneocyte and regulating the hydration and pH levels of the stratum corneum [28]. Epidermal dysfunction allow entry of allergens, which can subsequently lead to allergen sensitisation [29].

We also found a significant association between early wheeze and new inhalant allergen sensitization at 36 months. A possible mechanism is through the disruption of the airway epithelium that forms a physical barrier to entry of aeroallergens [30]. Asthmatic children may have a defective airway epithelial barrier with reduced E-cadherin [31]. E-cadherin contributes to the structural integrity of the airway epithelium. Disruption of E-cadherin may compromise the function of the epithelium in regulating the entry of aeroallergens, hence facilitating allergen sensitization [31].

The strengths of this study are the prospective study design with regular follow up and collection of data at multiple time points. In addition, skin prick testing is carried out at both 18–36 months which allow us to establish the temporal sequence between clinical phenotypes and allergen sensitization by looking at non-sensitized children at 18 months. However the main limitation of the study is the parental report of allergic outcomes which may have resulted in some misclassifications. The study team tried to overcome this by calling up the subjects to ascertain if the parental reports are accurate.

In conclusion, we identified clinical phenotypes that may predispose a child to developing allergen sensitization. Future research should assess whether improved clinical management of early-onset eczema and wheeze can reduce the risk of subsequent allergen sensitization.
